# Predicting Inpatient Falls Using Natural Language Processing of Nursing Records Obtained From Japanese Electronic Medical Records: Case-Control Study

**DOI:** 10.2196/16970

**Published:** 2020-04-22

**Authors:** Hayao Nakatani, Masatoshi Nakao, Hidefumi Uchiyama, Hiroyoshi Toyoshiba, Chikayuki Ochiai

**Affiliations:** 1 NTT Medical Center Tokyo Tokyo Japan; 2 Pharmaceutical Research Department Global Pharmaceutical R&D Division Neopharma Japan Co Ltd Tokyo Japan; 3 Research Development Department Lifescience AI Business Division FRONTEO Inc Tokyo Japan; 4 Tokyo Healthcare University Tokyo Japan

**Keywords:** fall, risk factor, prediction, nursing record, natural language processing, machine learning

## Abstract

**Background:**

Falls in hospitals are the most common risk factor that affects the safety of inpatients and can result in severe harm. Therefore, preventing falls is one of the most important areas of risk management for health care organizations. However, existing methods for predicting falls are laborious and costly.

**Objective:**

The objective of this study is to verify whether hospital inpatient falls can be predicted through the analysis of a single input—unstructured nursing records obtained from Japanese electronic medical records (EMRs)—using a natural language processing (NLP) algorithm and machine learning.

**Methods:**

The nursing records of 335 fallers and 408 nonfallers for a 12-month period were extracted from the EMRs of an acute care hospital and randomly divided into a learning data set and test data set. The former data set was subjected to NLP and machine learning to extract morphemes that contributed to separating fallers from nonfallers to construct a model for predicting falls. Then, the latter data set was used to determine the predictive value of the model using receiver operating characteristic (ROC) analysis.

**Results:**

The prediction of falls using the test data set showed high accuracy, with an area under the ROC curve, sensitivity, specificity, and odds ratio of mean 0.834 (SD 0.005), mean 0.769 (SD 0.013), mean 0.785 (SD 0.020), and mean 12.27 (SD 1.11) for five independent experiments, respectively. The morphemes incorporated into the final model included many words closely related to known risk factors for falls, such as the use of psychotropic drugs, state of consciousness, and mobility, thereby demonstrating that an NLP algorithm combined with machine learning can effectively extract risk factors for falls from nursing records.

**Conclusions:**

We successfully established that falls among hospital inpatients can be predicted by analyzing nursing records using an NLP algorithm and machine learning. Therefore, it may be possible to develop a fall risk monitoring system that analyzes nursing records daily and alerts health care professionals when the fall risk of an inpatient is increased.

## Introduction

### Background

Falls are the most common risk factor affecting the safety of hospital inpatients. They often result in a severe injury, such as a femoral fracture or head trauma, which can be life-threatening or affect the patient’s quality of life. After analyzing data from 1263 hospitals, Bouldin et al [1] reported that the rate of falls in the United States was 3.56 per 1000 patient-days during a 27-month study period and that 26.1% of these falls (0.93 per 1000 patient-days) resulted in injury. In Japan, a 2016 report from the Japan Federation of Democratic Medical Institutions indicated that the rates of falls and falls causing injury were 4.40 and 0.29 per 1000 patient-days, respectively [2]. Therefore, the prevention of falls is one of the most important areas of risk management for health care organizations. The Joint Commission, which is involved in the accreditation and certification of US health care organizations and programs, has strongly recommended taking strategic action for fall prevention, including the use of a standardized assessment tool to identify risks [3].

### Prior Work

A variety of methods have been developed to predict the risk of falls for hospital inpatients, such as the Morse Fall Scale [4], St Thomas’s Risk Assessment Tool in Falling Elderly Inpatients (STRATIFY) [5], Hendrich Fall Risk Model (HFRM) [6], and the revised Hendrich II Fall Risk Model [7]. All these methods have been used and evaluated [8-11]. However, such risk assessment methods invariably involve time-consuming processes, such as interviews, observation, and intervention [4-7], which interrupt the work of health care professionals, and the additional workload contributes to an increase in medical costs.

Moreover, several studies, including systematic reviews, have demonstrated that no single intervention, including patient tags and movement sensors, efficiently reduces fall incidents in any setting, whereas multifactorial assessment linked to appropriate interventions is successful [12-16]. However, no common combination of risk factors was discovered in these studies [17], indicating that health care professionals still need to conduct multiple assessments for each risk factor in daily practice, including motor function, continence, mental state, and medication. Thus, a less laborious assessment tool that can predict the risk of falls with high precision without initial intervention is desirable.

With recent advances in information technology, several groups have attempted to apply natural language processing (NLP) to text analysis of electronic medical records (EMRs) to achieve the early diagnosis of conditions such as peripheral arterial disease [18], asthma [19], and multiple sclerosis [20]. In these studies, NLP was used to find specific words or phrases in a predefined dictionary that described the symptoms or signs of each disease. Following these studies, we apply artificial intelligence to EMRs to analyze the risk of falls.

### Goal of This Study

Our primary objective is to determine whether hospital inpatient falls can be predicted through the analysis of the unstructured text of hospital nursing records in Japanese EMRs using an NLP algorithm and machine learning. In nursing records, nurses write daily information about a patient’s nursing care, the patient’s response, and other events or factors that may affect the patient’s well-being based on observation and experience [21]. Thus, nursing records contain valuable information for clinical practice but have not been widely used for any type of risk assessment because they require a technique, such as NLP, to analyze and extract meanings of interest from free text or unstructured documents.

We constructed a predictive model to assess the linguistic differences between the nursing records of fallers and nonfallers using our proprietary algorithm applying NLP in combination with machine learning and evaluated its performance using receiver operating characteristic (ROC) analysis. The advantages of our approach are that it allows us to assess various risk factors from a single input (nursing records), and it is less laborious and costly than previous approaches because it does not require additional observation or interviews.

## Methods

### Study Design

We used a case-control study because of the easy availability of nursing records in EMRs, limited computational capacity, and low rate of falls among inpatients. Because our main objective is to verify the feasibility of using nursing records to predict falls, we used only one hospital and one year of data to limit the cost and time of data extraction. For this study, we considered NTT Medical Center Tokyo (Tokyo, Japan), which is an acute hospital with 606 beds and an average hospital stay of 11.4 days. The Institutional Review Board of the hospital approved the study (Approval #15-267, June 25, 2015). The study period was from July 2014 to July 2015.

### Data

Among 18,045 inpatients during the study period, 335 patients with one or more fall incidents (fallers) were identified from the incident reports of the hospital. As a control group, 408 patients without falls (nonfallers) were randomly selected. More nonfallers than fallers were chosen as a contingency if extracted data had to be discarded for unexpected reasons. Data were not discarded; therefore, all usable data were considered in the analysis. We are aware that the substantial difference between the total number of fallers and nonfallers can affect machine learning; however, we believe this is mitigated by the use of a case-control study, which is often used in rare medical cases such as rare diseases.

Data on the two groups of patients were extracted from the EMR system by the EMR vendor and provided to the researchers after anonymization. The researchers constructed a case data set (fallers) and control data set (nonfallers). The nursing records were written in the EMR once a day or more frequently as necessary by several nurses using the subjective, objective, assessment, and plan style or free description. These contained (1) patients’ statements, (2) observations of the nurses, (3) results of vital check and various assessments, (4) descriptions of medical treatment and administration of drugs (or plan for them), (5) messages to and from patients, and (6) any other comments by nurses. Some parts of (3) and (4) were entered as preset form data, and others were unstructured data. Several records for one patient made on the same day were integrated into one nursing record. Thus, 25,145 nursing records were obtained, which consisted of 18,912 nursing records for fallers and 6233 for nonfallers. The prevalence of falls was 2.61 falls per 1000 patient-days during the study period. The characteristics of the patients and nursing records are shown in Table 1.

The entire nursing record data set was divided into a learning data set and test data set by generating random numbers for patient identification numbers assigned after anonymization.

**Table 1:** Characteristics of the patients and nursing records.

Characteristics		All patients	Fallers	Nonfallers	*P* value^a^
Patients, n (% of total)		743 (100)	335 (45.1)	408 (54.9)	—^b^
**Gender, n (% of total)**					—
	Female	342 (100)	156 (45.6)	186 (54.4)	
	Male	401 (100)	179 (44.6)	222 (55.4)	
Age (years), mean (SD)		67.0 (17.1)	73.3 (13.3)	65.5 (18.1)	<.001
Nursing records, n		25,145	18,912	6233	—
Nursing records per patient, mean (SD)		45.3 (43.5)	68.1 (49.1)	26.6 (26.4)	<.001
Nursing record length,^c^ mean (SD)		5392.1 (4138.2)	5628.4 (4202.6)	4675.1 (3848.8)	<.001

^a^Welch *t* test between fallers and nonfallers used.

^b^Not applicable.

^c^Number of Japanese or Chinese characters.

### Data Exclusion

The nursing records that did not satisfy the criterion of more than 50 Japanese or Chinese characters were excluded during tokenization and vectorization. This was a requirement of the Concept Encoder, which is described subsequently.

### Data Processing by Concept Encoder

A model was constructed to sort the nursing records into two groups (“risk” and “no risk”) from the learning data set. The probability of being categorized in the risk group, hereafter referred to as the risk probability, was calculated for each nursing record in the test data set using an in-house algorithm for NLP and machine learning called Concept Encoder (FRONTEO, Inc, Tokyo, Japan; will be published elsewhere), which was constructed on a Python platform.

### Document and Word Embedding

Concept Encoder performs text analysis by defining the line vector obtained from the document-word matrix as a document vector. First, each document is decomposed into morphemes (the smallest meaningful units of a language) by morphological analysis using MeCab version 0.996 [22], and rules are applied to label each element at the morpheme level with a word. Morphemes that were not words were discarded before each element was labeled. Then the word labels are embedded in *k*-dimensional vector space [23-25]. Documents can also be embedded in the *k*-dimensional vector space by expanding the word-embedding method. Assuming that there are *m* documents and *n* words in all the nursing records used in the study, and they are embedded, these documents and words can be expressed as matrices *D* and *W*:

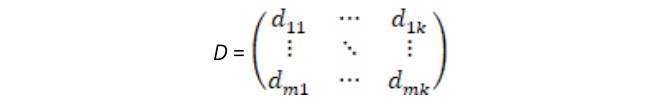

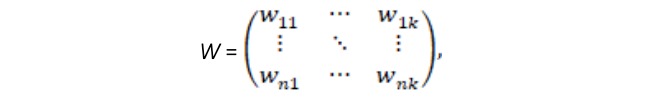





where each row vector of matrices *D* and *W* corresponds to *m* documents and *n* words, respectively, from the nursing records in the study.

It is well known that embedded vectors have interesting features, such as word analogy, and outperformed bag of words approaches in several linguistic tasks. These interesting features are retained after two matrices are multiplied because of the linearity of multiplication. For example, if 
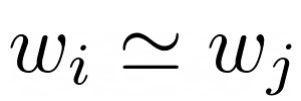
 for two row vectors in *W*, then the inner product with *d,* which is a row vector in matrix *D,* holds 
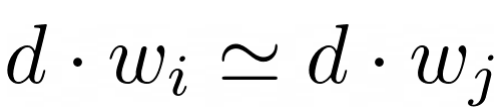
. Expanding this to the word analogy, if 
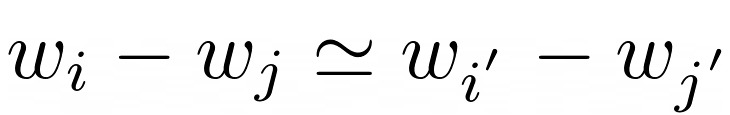
, where 
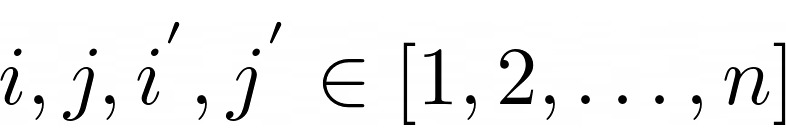
 holds for four row vectors in *W*, then 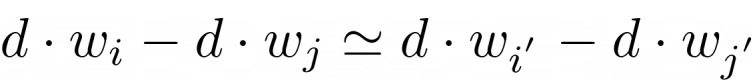 holds for any row vector *d* in *D*. Hence, the product of these two matrices generates the *DW* matrix, which is a document-word matrix that also has these interesting features:

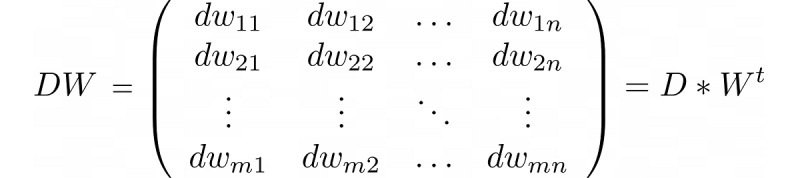



As seen in previous studies [23-25], neural networks have been used to calculate *D* and *W,* and if the number of documents becomes large, then the calculation of these matrices is computationally intensive. Hence, the words included in the neural embedding are restricted to the top 1000 most popular words that occur in the documents in the learning data set, hereafter referred to as the “top 1000 words.”

In this study, for *W,* the skip-gram with the negative sampling algorithm was used. The hyperparameter number of negative sampling was set to 5, and the number of dimensions for *W* was set to 300. For *D,* the distributed bag of words version of the paragraph vector (PV-DBOW) was used with the same negative sampling and embedding dimensions as *W.* After obtaining *W* and *D,* the *DW* matrix was calculated using matrix multiplication.

### Construction of the Fall Prediction Model

For the construction of the fall prediction model, the *DW* matrix was derived from all documents and words in the learning data set. By attaching tags of 1 (for fallers) and 0 (for nonfallers) to each document, each line vector of the *DW* matrix (which corresponds to *m* documents) was associated with a tag of 0 or 1. Each word was subjected to adaptive weighting for optimum separation between fallers and nonfallers using a logistic regression model, and the weighted parameters were estimated by the Markov chain Monte Carlo (MCMC) method with a normal distribution as the prior distribution of weights. For the MCMC approach, the weighted parameters were estimated using posterior distributions, and uncertainty of the estimate was also considered by observing the distribution. The weighted parameters thus obtained were used as the fall prediction model to evaluate the test data set. Random bisection of the learning data set was conducted three times, and six models were constructed using the six bisected data sets. Because the sample size was not balanced between fallers and nonfallers, we used the synthetic minority oversampling technique in this step [26] by using the function of “imblearn.over_sampling.SMOTE” from the library [27] with the default setting and checked that samples for not majority class (“faller” or “imminent”) were resampled to be equal to those the major one in number. Morphemes that significantly contributed to the separation of the fallers and nonfallers in at least four of the six primary models (ie, “significant vocabulary”) were extracted and were used to construct the final model by the generation of the trimmed *DW* matrix followed by MCMC optimization.

### Evaluation of Documents in the Test Data Set

For evaluation, documents in the test data set were tokenized to generate another matrix (hereafter called “*DW* for test”) using the top 1000 words followed by trimming it down using the significant vocabulary. The risk probability was calculated as the element-wise product of the corresponding line vector of the *DW* for test matrix and the final model. To assess the significance of differences, the Student *t* test was performed using R studio software (version 1.0.143).

## Results

### Analysis of the Data Set

Differences were observed between the groups of fallers and nonfallers for age, number of nursing records per patient (strongly correlated with the duration of hospitalization), and the length of nursing records (Table 1; *P*<.001 by Welch *t* test). The ratios of fallers and nonfallers also varied among some clinical divisions of the hospital, as shown in Table 2. However, matching for such factors was not performed because our primary aim was to determine whether it was possible to predict falls through comprehensive risk assessment using text analysis of nursing records regardless of risk factors already known or presumed from other information.

**Table 2:** Number of inpatients per clinical division.

Clinical division	Total (N=743), n	Fallers (n=335), n	Nonfallers (n=408), n
Gastroenterology	107	51	56
Surgery	104	42	62
Cardiology	53	22	31
Gynecology and obstetrics	49	4	45
Stroke unit	44	27	17
Orthopedic surgery	41	23	18
Respirology	37	20	17
Urology	36	12	24
Hematology	32	27	5
Neurosurgery	31	19	12
Psychiatry	30	23	7
Pain clinic	27	10	17
Otorhinolaryngology	21	1	20
Medical cooperation	17	7	10
Nephrology	16	9	7
Dermatology	16	3	13
Ophthalmology	15	4	11
Palliative care	14	9	5
Gamma knife center	13	1	12
Dentistry and oral surgery	9	3	6
General thoracic surgery	8	4	4
Neurology	8	6	2
Emergency medicine	5	5	0
Cardiovascular surgery	4	2	2
Endocrinology and metabolism	3	0	3
General medicine	2	0	2
Psychosomatic medicine	1	1	0

### Model to Predict Falls

The entire data set was divided into a learning data set and test data set as shown in Table 3. To construct a model to predict falls, tokenization and vectorization were performed on the learning data set. During this step, 12 nursing records (five for fallers and seven for nonfallers) that did not contain more than 50 Japanese or Chinese characters were excluded, leaving 9094 nursing records for fallers and 3513 nursing records for nonfallers. Using NLP and machine learning for the unstructured text of the learning data set, 378 morphemes that corresponded to significant vocabulary (ie, they contributed to separating fallers from nonfallers in at least four of the six primary models) were selected (a partial list is shown in Textbox 1). To construct the final model, 378 columns that corresponded to the selected morphemes were extracted from the 1000 columns of the *DW* matrix generated using the learning data set and were again subjected to optimization to separate fallers from nonfallers using the MCMC method. Using the final model, the probability of each nursing record in the test data set being in the risk category was evaluated next.

**Table 3:** Characteristics of patients and nursing records in the learning data set and test data set for prediction of falls.

Entire data set			Total	Fallers	Nonfallers	*P* value^a^
**Learning data set**						
	Patients, n (% of total)		371 (100)	167 (45.0)	204 (55.0)	—^b^
	**Gender, n (% of total)**					—
		Female	159 (100)	78 (49.1)	81 (50.1)	
		Male	212 (100)	89 (42.0)	123 (58.0)	
	Age (years), mean (SD)		67.0 (17.0)	73.4 (12.9)	61.7 (18.1)	<.001
	Nursing records, n		12,619	9099	3520	—
	Nursing records per patient, mean (SD)		45.4 (41.9)	66.4 (45.3)	28.2 (29.3)	<.001
	Nursing record length^c^, mean (SD)		4879.1 (2212.3)	5559.4 (1961.9)	4323.8 (2090.9)	<.001
**Test data set**						
	Patients, n (% of total)		372 (100)	168 (45.2)	204 (54.8)	—
	**Gender, n (% of total)**					—
		Female	183 (100)	78 (42.6)	105 (57.4)	
		Male	189 (100)	90 (47.6)	99 (52.4)	
	Age (years), mean (SD)		67.1 (17.1)	73.2 (13.8)	62.1 (18.1)	<.001
	Nursing records, n		12,526	9813	2713	—
	Nursing records per patient, mean (SD)		45.2 (45.1)	69.8 (52.6)	25.0 (23.0)	<.001
	Nursing record length,^c^ mean (SD)		4739.6 (2127.5)	5522.9 (2005.8)	4094.5 (2009.1)	<.001

^a^Welch *t* test between fallers and nonfallers used.

^b^Not applicable.

^c^Number of Japanese or Chinese characters.

**Morphemes used in the model for predicting falls. Morphemes related to known or potential risk factors (indicated in brackets) were extracted from 378 morphemes used in the final model of the first experiment.**

**[Psychotropics]**

- Seroquel, Lendormin, Serenace

**[Mental status]**

- recognition, dementia, arousal, mental status, somnolence willingness, cognitive function, orientation, esthesia, sleeplessness, anxiousness, Myslee

**[Motor function]**

- postural change, aid, assistance, support, lower limb, rehabilitation, slippers, wheelchair, sitting square, torpor, self-standing, parallel bars, limb, daily life behavior, lumbar region, ride, body posture, dorsal region, gait, extension (of limbs), walking stick

**[Excretion]**

- excretion, defecation, constipation, incontinence, Lasix, Pursennid, Biofermine

**[Oropharyngeal]**

- mouth, sputum, hospital food, oral, water drinking, nausea, swallowing, vomiting, dentures, fluid, mouth rinse, eat

**[Circulation]**

- WBC (white blood cells), blood pressure, transfusion, anemia, mmHg, oxygen, neutrophil, blood, pulse, vein, bleeding, blood vessel, heartbeat, platelet

Similar to the process used for the learning data set, nursing records with fewer than 50 characters (13 and 4 nursing records for fallers and nonfallers, respectively) were deleted from the test data set, leaving 9800 nursing records for fallers and 2709 nursing records for nonfallers. For each patient in the test data set, the mean value of the risk probabilities for all their nursing records was calculated as a patient risk score that was used to evaluate the performance for predicting falls by ROC analysis. To draw the ROC curve, we calculated the true positive rate and false positive rate using the patient risk score (continuous variables that range from 0 to 1) and category (faller or nonfaller) for each patient. Scanning the cutoff values from 0 to 1, the true and false positive rates were calculated from the confusion matrix for each cutoff value.

**Figure 1:**
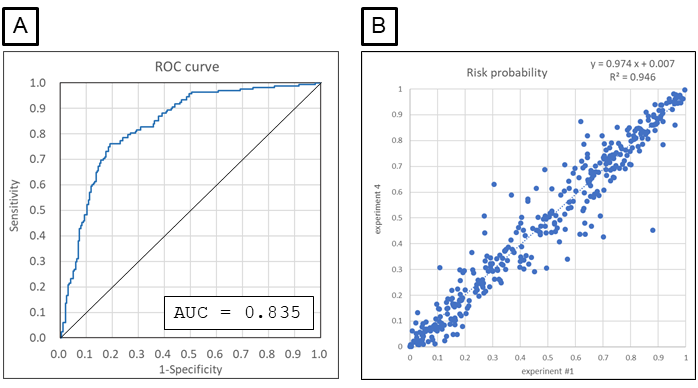
Precision and reproducibility of the model for predicting falls using the test data set. Five independent experiments were conducted for the learning and testing steps. A: receiver operator characteristic (ROC) curve for experiment 1; B: scatterplot of patient risk scores for two of the five experiments (1 and 4). AUC: area under the curve.

As shown in Figure 1A, the area under the ROC curve (AUC) was 0.835, which indicates excellent separation between fallers and nonfallers. Applying a threshold score of 0.5602, corresponding to the point on the ROC curve closest to the coordinate (0, 1), each patient was sorted into risk and no risk categories, as shown in the confusion matrix (Table 4). Then the sensitivity, specificity, and odds ratios were calculated (Table 5). Sensitivity and specificity are the most commonly used measures for diagnostic performance from the viewpoint of actual medical practice, in which the former is the rate of correct diagnosis among all disease patients and the latter is the rate of correct diagnosis among all normal patients. The odds ratio is the most commonly used measure in case-control studies.

Next, the reproducibility of the analysis was examined by conducting similar experiments four more times (experiments 2 to 5). The model was constructed with a new learning data set, and the test data set was evaluated by generating random numbers for patient identification numbers, after which scatterplots were drawn to check correlations of patient risk scores between all combinations of two experiments (an example for experiments 1 and 4 is shown in Figure 1B). The analytical indexes for the five independent experiments demonstrated the high precision (Table 5) and reproducibility (Figure 1B and Table 6) of the model for the prediction of falls. These results demonstrated that text analysis of nursing records was an efficient method for predicting falls with high reproducibility.

**Table 4:** Confusion matrix of fall prediction for experiment 1.

Prediction	Patients		
	Fallers, n	Nonfallers, n	Total, N
Risk	128	39	167
No risk	40	165	205
Total	168	204	372

**Table 5:** Reproducibility of the model for predicting falls. A summary of evaluation indexes for the five experiments are shown.

Statistic	Experiment					Mean (SD)
	1	2	3	4	5	
Area under the curve	0.835	0.831	0.832	0.842	0.831	0.834 (0.005)
Sensitivity (95% CI)	0.762 (0.714-0.813)	0.75 (0.702-0.801)	0.774 (0.726-0.824)	0.78 (0.730-0.823)	0.78 (0.732-0.830)	0.769 (0.013)
Specificity (95% CI)	0.809 (0.766-0.854)	0.794 (0.751-0.839)	0.779 (0.736-0.825)	0.789 (0.724-0.801)	0.755 (0.712-0.801)	0.785 (0.02)
Odds ratio (95% CI)	13.54 (8.23-22.27)	11.57 (7.11-18.83)	12.09 (7.40-19.73)	13.26 (8.07-21.78)	10.9 (6.72-17.71)	12.27 (1.11)

**Table 6:** Correlations (R^2^ for linear regression) of all combinations of two out of five experiments are shown.

Experiment	1	2	3	4	5
1	—	0.939	0.952	0.946	0.945
2	—	—	0.932	0.937	0.957
3	—	—	—	0.948	0.957
4	—	—	—	—	0.945
5	—	—	—	—	—

### Imminent Precursors of Falls

**Figure 2:**
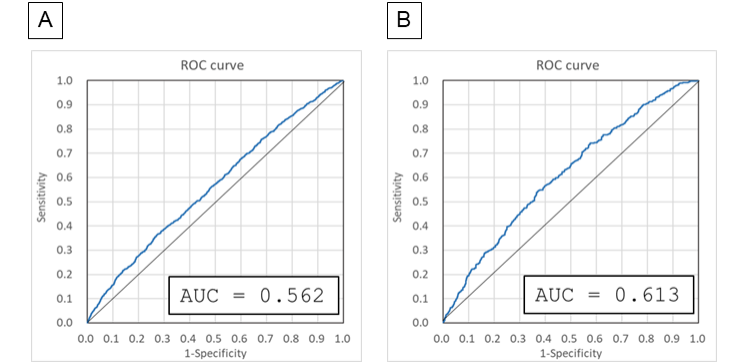
Precision of the model for detecting imminent precursors using the faller data set. Five independent experiments were conducted for the learning and testing steps to identify imminent precursors of falls among all fallers (A) and among fallers who were short-term patients (B). Receiver operating characteristic (ROC) curves for experiment 1 out of the five experiments are shown. AUC: area under the curve.

In the next step, the detection of the imminent precursors of falls was attempted by extracting specific features from the nursing records written several days before each incident. For the purpose, nursing records of all fallers were collected as “Faller data set” and then tagged with imminent (1-7 days before the fall) or not imminent (Table 7). After bisecting the faller data set into a learning data set and a test data set, the former was used to construct a model for discrimination of the tags by the same method described previously for risk/no risk categorization; that is, the final model was built from morphemes identified in at least four of the six primary models constructed using the learning data set. Then the final model was used to evaluate the probability of each faller nursing record in the test data set being placed in the imminent category, after which the performance of the detection of imminent precursors was evaluated using ROC analysis (Figure 2A) and the confusion matrix (Table 8). After four more independent examinations were performed in the same manner to check reproducibility, the average AUC of the ROC curve was 0.567 for the five experiments (Table 9), which demonstrates limited prediction of nursing records for imminent falls.

Based on the hypothesis that the medical conditions of long-term inpatients would be stable, and changes in risk factors for falls would be difficult to detect, we also performed separate analyses of long-term and short-term inpatients. Fallers with more than 60 nursing records or 45 or less nursing records were selected as long-term and short-term inpatients, respectively, and the prediction of imminent falls was conducted for each group (Table 7).

We found that improved prediction of imminent falls was achieved for short-term inpatients, with an AUC of mean 0.607 (SD 0.009) (for five independent experiments, Figure 2B and Tables 9 and 10), whereas prediction was poor for long-term inpatients (AUC mean 0.496, SD 0.011; summary table for the five experiments not shown). Confusion matrices were constructed for the short-term group, and the sensitivity, specificity, and odds ratios were calculated (Table 9). The results suggested that the calculated risk probability could be used to assess the imminent risk of falls for short-term inpatients at the time when each nursing record was written.

**Table 7:** Characteristics of patients and nursing records in the faller data set for detection of imminent precursors.

Faller data set			All fallers	>60 Nursing records	≤45 Nursing records
**Learning data set**					
	Patients, n		167	56	91
	**Gender, n**				
		Female	78	32	38
		Male	89	24	53
	Age (years), mean (SD)		73.4 (12.9)	74.7 (11.2)	73.0 (12.7)
	**Nursing records, n**		9094	5809	2231
		Imminent^a^	1114	487	464
		Not imminent	7980	5322	1767
	Nursing records per patient, mean (SD)		54.5 (45.7)	103.8 (45.7)	24.5 (12.3)
	Nursing record length, mean (SD)		5559.4 (1961.9)	5363.34 (1879.5)	5628.6 (2081.0)
**Test data set**					
	Patients, n		168	56	95
	**Gender, n**				
		Female	78	21	48
		Male	90	35	47
	Age (years), mean (SD)		73.2 (12.8)	72.4 (12.9)	74.0 (14.2)
	**Nursing records, n**		9813	6693	2239
		Imminent^a^	984	424	463
		Not imminent	8829	6269	1776
	Nursing records per patient, mean (SD)		58.4 (54.1)	119.5 (51.9)	23.6 (12.6)
	Nursing record length, mean (SD)		5522.9 (2005.8)	5022.2 (2187.5)	5662.8 (1890.6)

^a^Nursing records registered within seven days before a fall.

**Table 8:** Results of discrimination of imminent precursors of falls among all fallers. Confusion matrix for experiment 1 out of five experiments is shown.

Prediction	Nursing records		
	Imminent	Not imminent	Total
Imminent	553	4281	4834
Not imminent	429	4536	4965
Total	982	8817	9799

**Table 9:** Reproducibility of the model for detecting imminent precursors using the faller data set. Five independent experiments were conducted for the learning and testing steps to identify imminent precursors of falls among all fallers and among fallers who were shot-term patients.

Group and statistic		Experiment					Mean (SD)
		1	2	3	4	5	
**Fallers**							
	Area under the curve	0.562	0.576	0.568	0.566	0.564	0.567 (0.005)
	Sensitivity (95% CI)	0.563 (0.546-0.581)	0.543 (0.526-0.560)	0.611 (0.593-0.630)	0.576 (0.559-0.594)	0.536 (0.519-0.553)	0.566 (0.030)
	Specificity (95% CI)	0.514 (0.509-0.520)	0.576 (0.571-0.582)	0.477 (0.472-0.482)	0.517 (0.512-0.522)	0.558 (0.552-0.563)	0.529 (0.039)
	Odds ratio (95% CI)	1.37 (1.20-1.56)	1.62 (1.42-1.84)	1.43 (1.25-1.64)	1.46 (1.27-1.66)	1.45 (1.27-1.66)	1.47 (0.09)
**Fallers who were short-term patients**							
	Area under the curve	0.613	0.607	0.595	0.602	0.618	0.607 (0.009)
	Sensitivity (95% CI)	0.547 (0.522-0.572)	0.649 (0.621-0.677)	0.492 (0.470-0.515)	0.607 (0.581-0.635)	0.623 (0.596-0.651)	0.584 (0.063)
	Specificity (95% CI)	0.626 (0.613-0.641)	0.524 (0.512-0.536)	0.653 (0.639-0.668)	0.548 (0.535-0.560)	0.560 (0.547-0.573)	0.582 (0.055)
	Odds ratio (95% CI)	2.02 (1.64-2.49)	2.03 (1.64-2.51)	1.83 (1.48-2.25)	1.87 (1.52-2.31)	2.10 (1.70-2.59)	1.97 (0.12)

**Table 10:** Results of discrimination of imminent precursors of falls among fallers who were short-term patients. Confusion matrix for experiment 1 out of five experiments is shown.

Prediction	Nursing records		
	Imminent	Not imminent	Total
Imminent	252	663	915
Not imminent	209	1112	1321
Total	461	1775	2236

## Discussion

### Principal Results

Our results confirmed it is possible to predict inpatient falls using text analysis of nursing records in a hospital EMR system, with an AUC of 0.834 across an average of five independent experiments. In many previous studies, the prediction of falls was based on specified risk factors, such as the use of psychotropic drugs [28-32], mental state (eg, disorientation, confusion, and delirium) [4,5,30,33-35], impaired motor function (eg, unstable gait and muscle weakness) [4,5,29,32,35], and excretory condition (eg, incontinence and frequent toileting) [5,33,35]. Additionally, the usefulness of nursing records for inpatient fall prediction was discussed recently [36], and it was shown that nursing records contained words known as risk factors for inpatient falls and interventions used in daily practice using NLP analysis. However, all the words identified in the analysis were preselected using prior reports, risk assessment tools, and subject matter expert’s knowledge. By contrast, we did not focus on any specific factor or emphasize any specific keywords, topics, concepts, or fields throughout our NLP analysis of unstructured text in nursing records and subsequent machine learning. Despite this, we found many words closely related to the previously mentioned risk factors in the list of morphemes that contributed to the prediction of fall risk (Textbox 1). Thus, the Concept Encoder successfully extracted known risk factors for falls as words with a statistically significant correlation to actual incidents. It is possible that several other words (or related concepts) that contribute to the model might be unknown risk factors. These candidate novel risk factors may not only be useful for predicting falls but also for determining the causes of falls or selecting interventions for prevention. In future work, we will conduct further numerical analyses of these candidates to examine their similarities or relationships, such as cluster analysis or context analysis based on the document-word embedding matrix (*DW*). If it is proven that words related to known and novel risk factors are effective for predicting falls, this might encourage hospital nurses to write nursing records that emphasize these factors, thus improving the quality of nursing records and allowing falls to be predicted with higher precision.

There was a statistically significant difference between nursing records recorded one to seven days before a fall and others. This suggests that a fall risk monitoring system designed to analyze nursing records daily and alert health care professionals when an increase of fall risk is detected could be an effective tool for the prevention of falls. Recently, the authors developed a new version of Concept Encoder with improved computational capacity and deployed for a currently ongoing study using a larger data set (all nursing records for three years; approximately 520,000 nursing records from 900 fallers and 28,000 nonfallers). Encouraged by the early results of the study, which has shown considerable improvement in the prediction for imminent falls (AUC of approximately 0.73), the authors have developed the first version of the fall risk monitoring system.

Because nursing records contain continuous information covering a broad context regardless of the underlying disease or complications and results of various medical tests and vital signs, this algorithm can be applied to construct models for predicting other specific medical interests, such as a sudden change of the patient’s condition or recurrence of acute illness. It also has the potential to be used as the basis of a multipurpose diagnosis and caregiving support system.

Recent developments in machine learning technology have enhanced the range of application, but it is still rarely used in the health care field. One reason is that neural network analysis, such as deep learning, cannot provide human-interpretable models or rules because of the numerous layers in the learning process. This “black box problem,” that is, poor traceability of the learning and analysis processes, is one reason that machine learning has not been widely applied in the health care field. The algorithm that we used (Concept Encoder) achieves very efficient transformation from documents to a document-word matrix, after which even simple logistic regression analysis can successfully predict falls. Moreover, the characteristics and probability distribution of the data are provided in an interpretable manner. Thus, even after a machine learning process is used, it can perform statistical analyses with high levels of stability, reproducibility, and verifiability that are required in the health care field. In this field, evidence-based decision making is valued, and vast amounts of medical data have been accumulated over many years for this purpose. It seems possible that Concept Encoder can be applied to mine these precious assets with verifiable analysis.

### Limitations

The low quantity of data may be a limitation in this study. However, due to the oversampling technique that we used, in which minority data were resampled to balance the two-group data set, we believe that the results of the study were not substantially affected by the low rate of falls. However, meta-analysis and a multicenter study will be considered in future work, which will generate more data. Additionally, we defined imminent as one to seven days before the fall. When we considered shorter time periods, such as one to three or one to five days before the fall, this reduced the number of imminent nursing records, which resulted in poorer prediction. In future work, larger data sets will enable the analysis of shorter time periods. Finally, as this is the first study to analyze nursing records using NLP and machine learning, there is no prior work available for comparison.

### Conclusions

We verified that text analysis of a single input—nursing records—using an NLP algorithm and machine learning was effective for the prediction of falls among hospital inpatients and the detection of imminent precursors of fall incidents. The approach was also able to extract useful information related to various types of fall risk factors, whether they are known or unknown, from the unstructured description of the nursing records. This can serve as a basis for a fall risk monitoring system (eg, screen-based) that can output risk factors for each high-risk patient together with the risk probability. We have already developed a prototype monitoring system and plan to start testing in collaboration with several hospitals. We are also developing an English version of our system for testing in English-speaking countries. Studies have reported that intervention is more successful when various health care professionals are involved as a team rather than taking a nursing-centric approach [17,37,38]. Thus, the output of data and risk factors provided by the system could be helpful for information sharing among teams of health care professionals at safety huddles or during handover.

## Abbreviations

AUC: area under the curve
EMR: electronic medical record
HFRM: Hendrich Fall Risk Model
MCMC: Markov chain Monte Carlo
NLP: natural language processing
PV-DBOW: paragraph vector-distributed bag of words
ROC: receiver operating characteristic
STRATIFY: St Thomas’s Risk Assessment Tool in Falling Elderly Inpatients
